# Supplementary material for: Therapeutic implications of altered cholesterol homeostasis mediated by loss of CYP46A1 in human glioblastoma
Source: EMBO Mol Med. 2019 Nov 28;12(1):e10924. doi: 10.15252/emmm.201910924 (PMC6949512; doi:10.15252/emmm.201910924)
Supplement: Supplementary file 1 — Appendix [file EMMM-12-e10924-s001.pdf]

Appendix

Therapeutic implications of altered cholesterol homeostasis mediated by loss of CYP46A1 in human glioblastoma

Mingzhi Han[1,2]*, Shuai Wang[1]*, Ning Yang[1], Xu Wang[1], Wenbo Zhao[1], Halala Sdik Saed[2], Thomas Daubon[3,4], Bin Huang[1], Anjing Chen[1], Gang Li[1], Hrvoje Miletic[2,5], Frits Thorsen[2,6], Rolf Bjerkvig[2,7#], Xingang Li[1#], Jian Wang[1, 2#]

Table of contents

Appendix Figure S1……………………………………………………………………………..2

Appendix Figure S2……………………………………………………………………………..3

Appendix Figure S3……………………………………………………………………………..5

Appendix Figure S4…………………………………………………………………………......6

Appendix Figure S5…………………………………………………………………………......7

Appendix Figure S6…………………………………………………………………………......8

Appendix Figure S7…………………………………………………………………………...10

Appendix Figure S8…………………………………………………………………………...11

Appendix Figure S9…………………………………………………………………………...12

Appendix Figure S10…………………………………………………………………………14

Appendix Table S1……………………………………………………………………………15

Appendix Table S2……………………………………………………………………………17

Appendix Table S3……………………………………………………………………………24

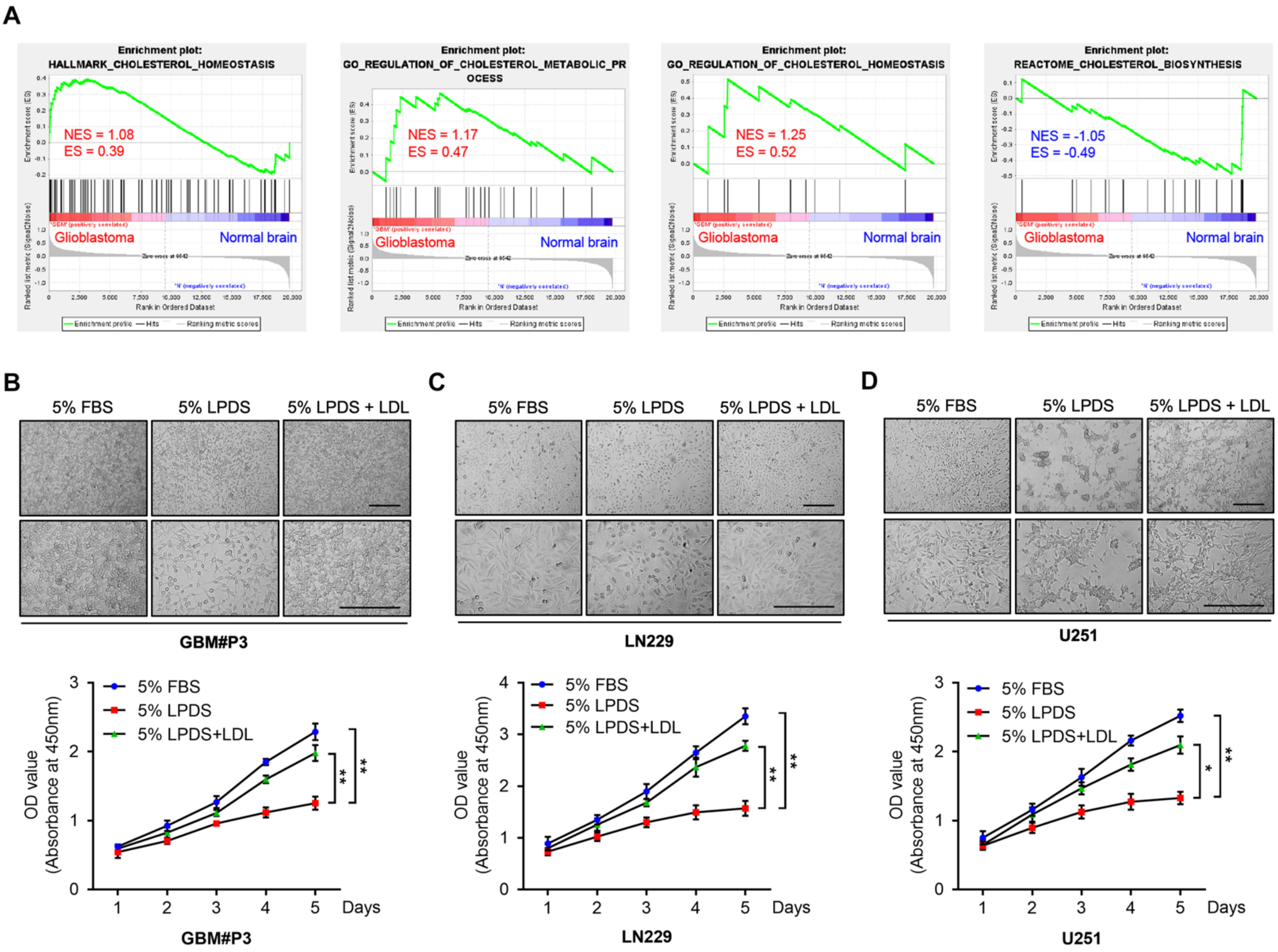

Figure S1. (A) GSEA plots show normalized enrichment score (NES) for hallmark cholesterol homeostasis, regulation of cholesterol metabolic process, regulation of cholesterol homeostasis and reactome cholesterol biosynthesis signatures in human GBM (*n* = 217) and normal brain (*n* = 28) using the Rembrandt dataset. (B-D) CCK8 cell growth curves and morphological differences of (B) GBM#P3, (C) LN229 and (D) U251 cells cultured in medium with 5% FBS or 5% lipoprotein deficient serum (LPDS) with or without low density lipoprotein (LDL, 5 μg/mL) for 5 days. Scale bar = 100 μm. Data are shown as the mean ± SEM (*n* = 3). GBM#P3: ***P* = 0.0013, ***P* = 0.0011; LN229: ***P* = 0.0035, ***P* = 0.0014; U251: **P* = 0.034, ***P* = 0.0011 (one-way ANOVA).

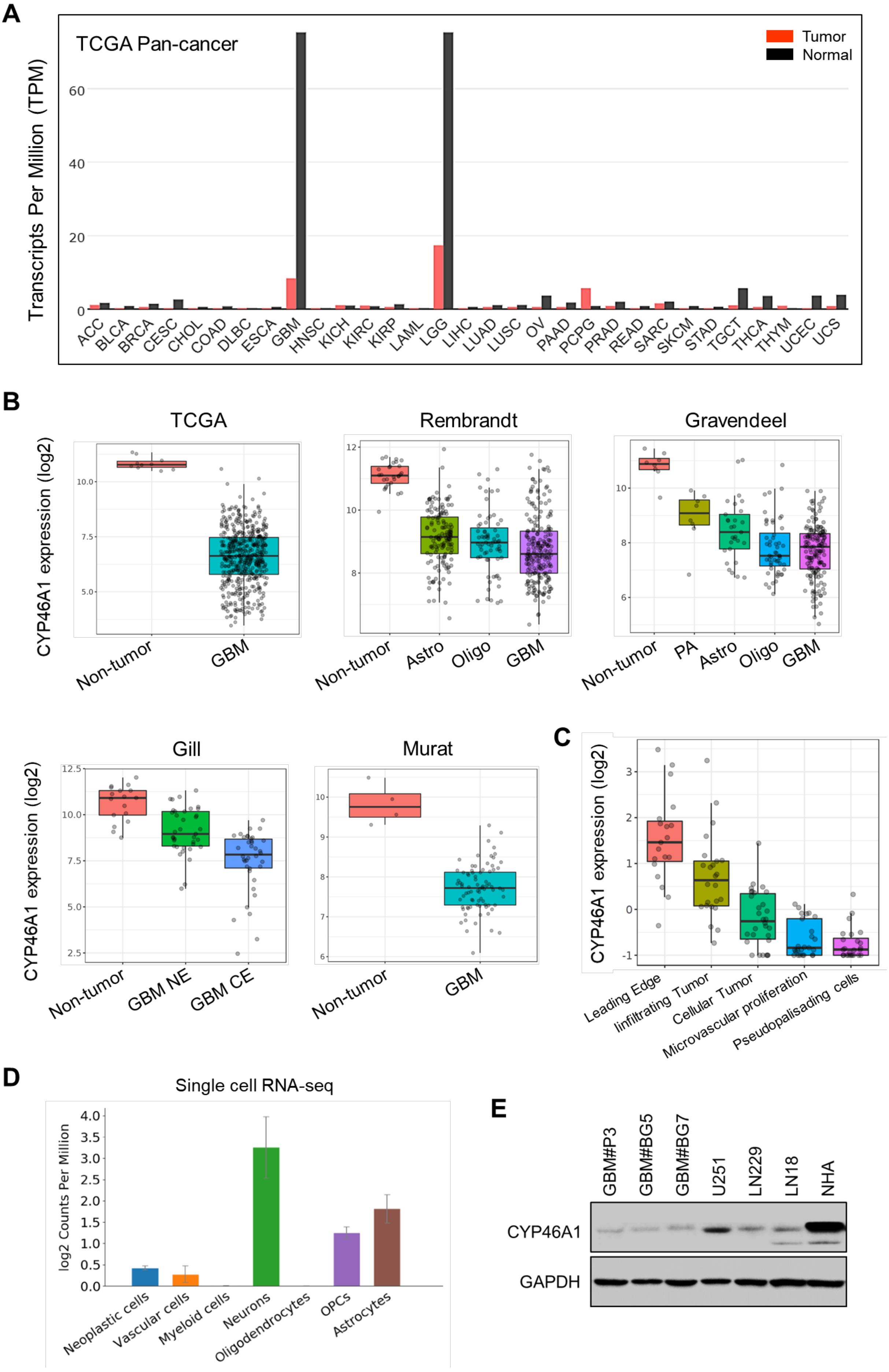
A
TCGA Pan-cancer
Tumor
Normal
Transcripts Per Million (TPM)
0
20
40
60
ACC BLCA BRCA CESC CHOL COAD DLBC ESCA GBM HNSC KICH KIRC KIRP LAML LGG LIHC LUAD LUSC OV PAAD PCPG PRAD READ SARC SKCM STAD TGCT THCA THYM UCEC UCS
B
TCGA
Rembrandt
Gravendeel
CYP46A1 expression (log2)
Non-tumor
GBM
Astro
Oligo
PA
Gill
Murat
GBM NE
GBM CE
C
CYP46A1 expression (log2)
Leading Edge
Iinfiltrating Tumor
Cellular Tumor
Microvascular proliferation
Pseudopalisading cells
D
Single cell RNA-seq
log2 Counts Per Million
Neoplastic cells
Vascular cells
Myeloid cells
Neurons
Oligodendrocytes
OPCs
Astrocytes
E
GBM#P3
GBM#BG5
GBM#BG7
U251
LN229
LN18
NHA
CYP46A1
GAPDH

Figure S2 (A) Expression levels of *CYP46A1* in 31 different TCGA cancer types and their matched normal tissues. (B) Box plots derived from 5 different datasets showing high expression levels of *CYP46A1* in normal brain compared to GBM tissues (Astro = astrocytoma, Oligo = Oligodendroglioma, PA = pilocytic astrocytoma, GBM NE = GBM non-enhancing, GBM CE = GBM contrast enhancing). (C) Intra-tumour heterogeneous expression characteristics of *CYP46A1* in specific anatomic structures obtained using IVY GBM RNA-seq data. (D) Single cell RNA-seq analysis from GSE84465 revealing high expression of *CYP46A1* in neurons, OPCs and astrocytes compared to neoplastic or vascular cells. Data are shown as the mean ± SEM. (E) Western blot analysis showing expression levels of CYP46A1 across different cell lines.

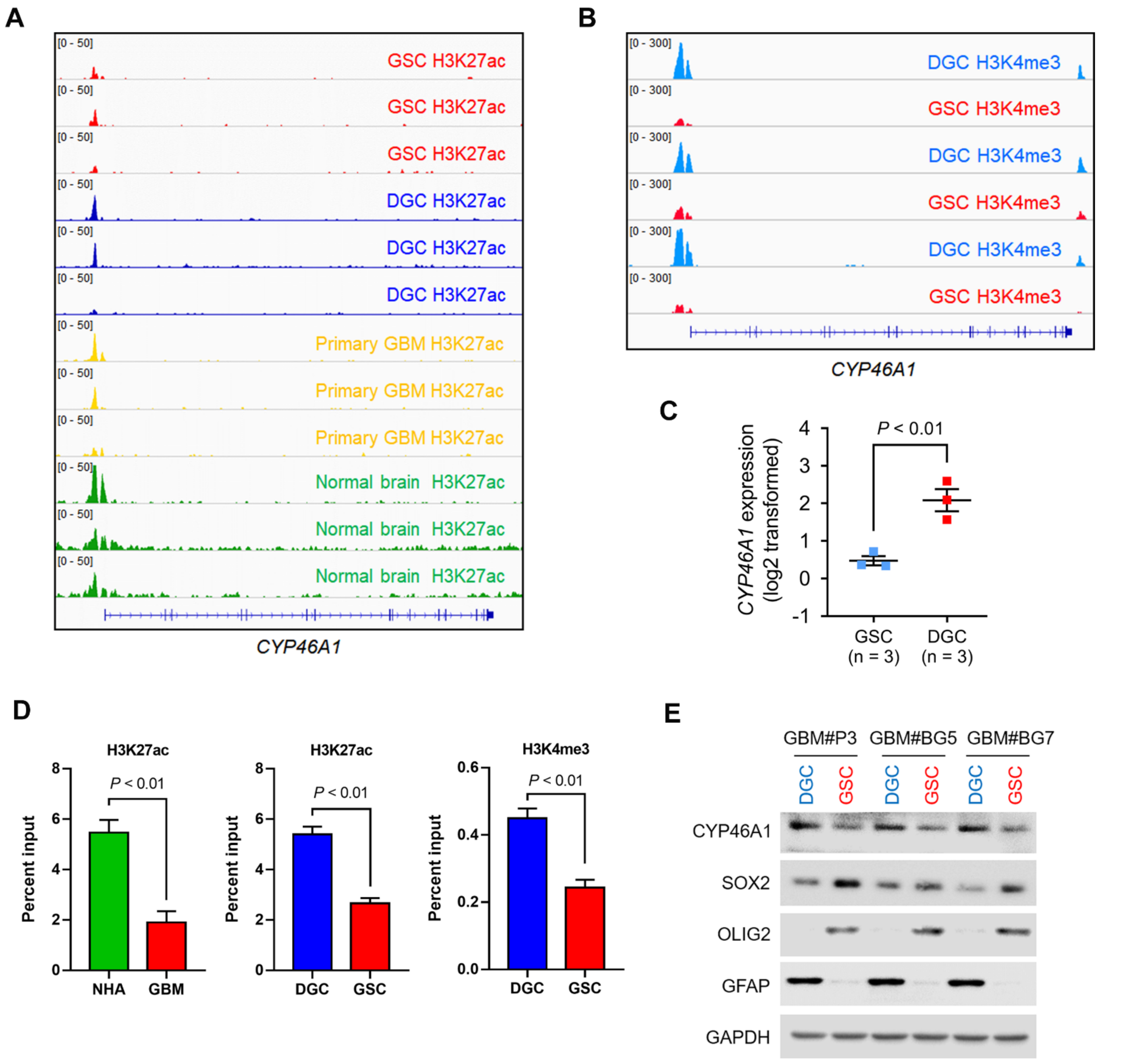

Figure S3 (A-B) Peak plots of H3K27ac and H3K4me3 in the promoter region of the *CYP46A1* locus in three matched pairs of GSCs and DGCs, primary GBMs, and normal brain tissue. (C) mRNA expression levels of *CYP46A1* in GSCs and DGCs. ChIP-seq and RNA-seq data were derived from GSE54047, GSE46016 and ENCODE databases. (D) ChIP-qPCR assay to detect the levels of H3K4me3 and H3K27ac in the promoter of *CYP46A1* in GBM cells. Data are shown as the mean ± SEM (n = 3). Statistical significance was determined by two-sided Student's t-test. (E) Western blot showing the differences in CYP46A1 protein levels between GSCs and serum-induced DGCs.

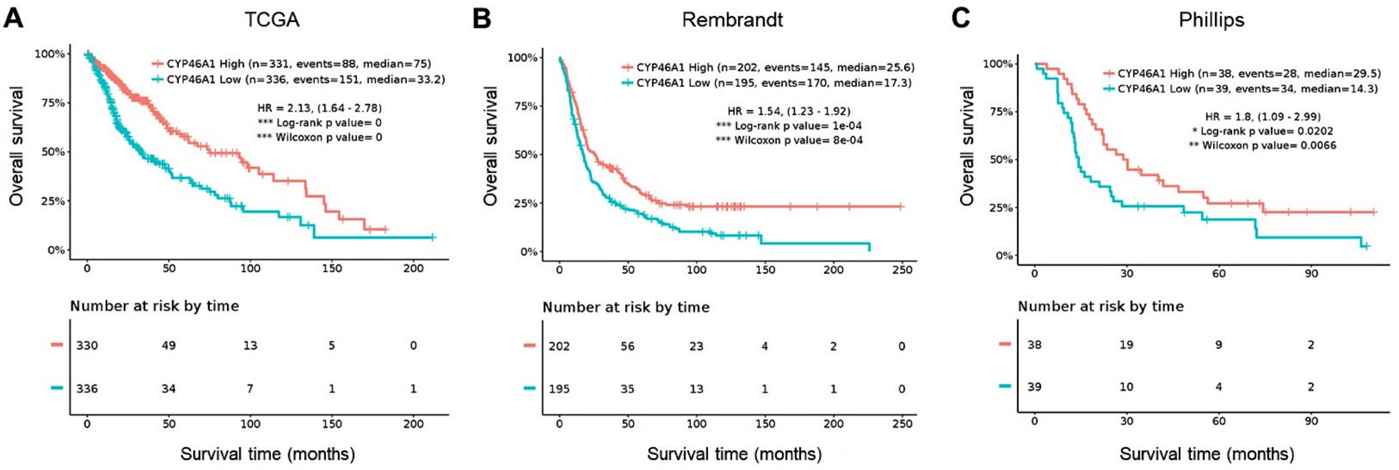

Figure S4 Kaplan-Meier analysis of patient OS based on high *vs* low expression of *CYP46A1* in TCGA (A), Rembrandt (B), and Phillips (C) datasets. *P*-values were obtained using the Log-rank test and Wilcoxon test.

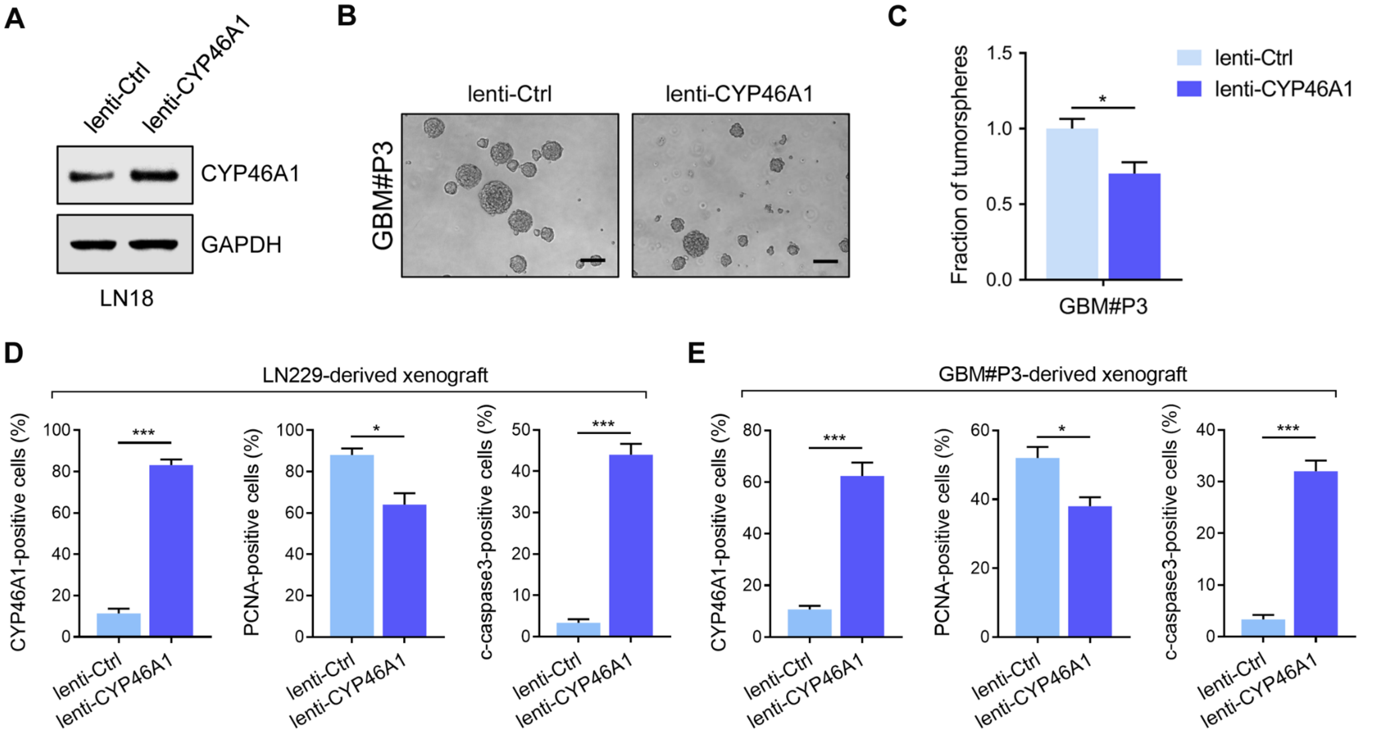

Figure S5 (A) Western blot to detect CYP46A1 in LN18 cells transduced with lenti-Ctrl or lenti-CYP46A1. GAPDH was used as the loading control. (B) Representative images of tumoursphere formation assays for GBM#P3 GSCs transduced with lenti-Ctrl or lenti-CYP46A1. Scale bar = 100 μm. (C) Graphic representation of the quantification of tumoursphere formation. Data are shown as the mean ± SEM. **P* = 0.039 (two-sided Student's t-test). (D-E) Graphic representation of the quantification of IHC staining in xenografts derived from (D) LN229: ****P* < 0.0001, **P* = 0.0197, ****P* = 0.0001; and (E) GBM#P3: ****P* = 0.0007, **P* = 0.0282, ****P* = 0.0002 (two-sided Student's t-test).

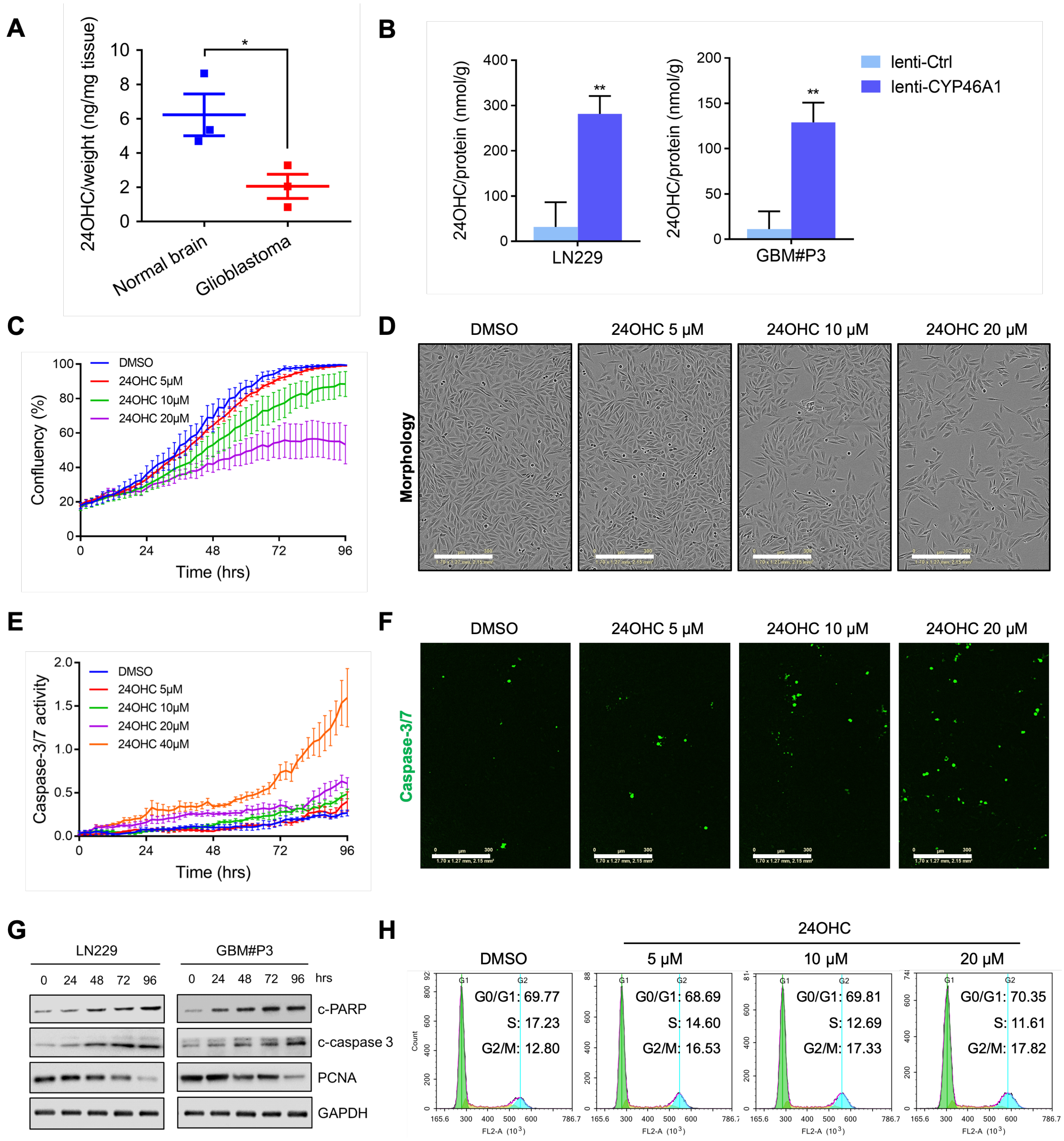

Figure S6 (A) 24OHC content in normal brain and GBM tissues (n = 3 per group). Data are shown as the mean ± SEM. *P = 0.0417 (two-sided Student's t-test). (B) Intracellular 24OHC levels in LN229 (left) and GBM#P3 (right) cells transduced with lenti-Ctrl or lenti-CYP46A1 measured using targeted LC-MS/MS (see Methods) and normalized to total protein. Data are shown as the mean ± SEM (n = 3). **P = 0.003, **P = 0.0023 (two-sided Student's t-test). (C) Growth curves for proliferation of LN229 cells treated with 24OHC (0 - 20 μM). Cell proliferation was measured using the IncuCyte proliferation assay. (D) Representative images of morphological differences of LN229 cells treated with 24OHC (0 - 20 μM) after 96 h. Scale bar = 300 μm. (E) Plots for caspase-3/7 activity over time to assess apoptosis in LN229 cells treated with 24OHC (0 - 40 μM). Caspase-3/7

activity was measured using the IncuCyte Apoptosis Assay. (F) Representative images of cleaved caspase-3/7 positive cells (green) in LN229 cells treated with 24OHC (0 - 20 μM) after 96 h. Scale bar = 300 μm. (G) Western blot to detect expression levels of cleaved PARP, cleaved caspase-3 and PCNA in LN229 and GBM#P3 after treatment with 20 μM 24OHC at the indicated time points. GAPDH was used as the loading control. (H) Flow cytometry for cell cycle analysis of PI stained LN229 cells after treatment with 24OHC (0 - 20 μM) for 48 h.

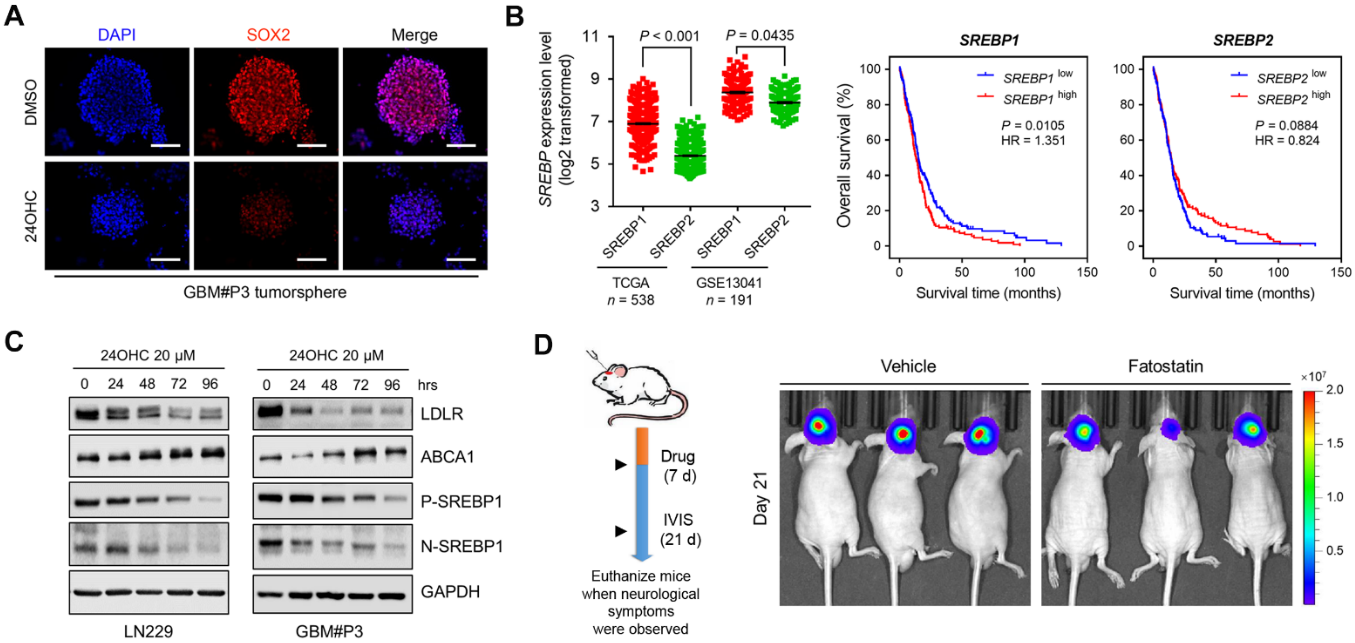

Figure S7 (A) Representative images of immunofluorescence staining for SOX2 (red) in GBM#P3 GSCs. Nuclei were counterstained with DAPI (blue). Scale bar = 50 μm. (B) *SREBP1* and *SREBP2* expression levels from TCGA and GSE 13041 datasets. Survival curves for GBM patients based on the expression of *SREBP1* and *SREBP2* from the TCGA dataset. (C) Western blot to detect expression levels of LDLR, ABCA1, P-SREBP1 and N-SREBP1 in LN229 and GBM#P3 cells after treatment with 20 μM 24OHC at the indicated time points. GAPDH was used as the loading control. (D) Bioluminescence images of mice orthotopically implanted with luciferase-expressing GBM#P3 cells (*n* = 3 per group). Mice were randomly divided into vehicle control (sterile PBS) or fatostatin (15 mg/kg) groups with intraperitoneal injection every day 7 days after orthotopic implantation.

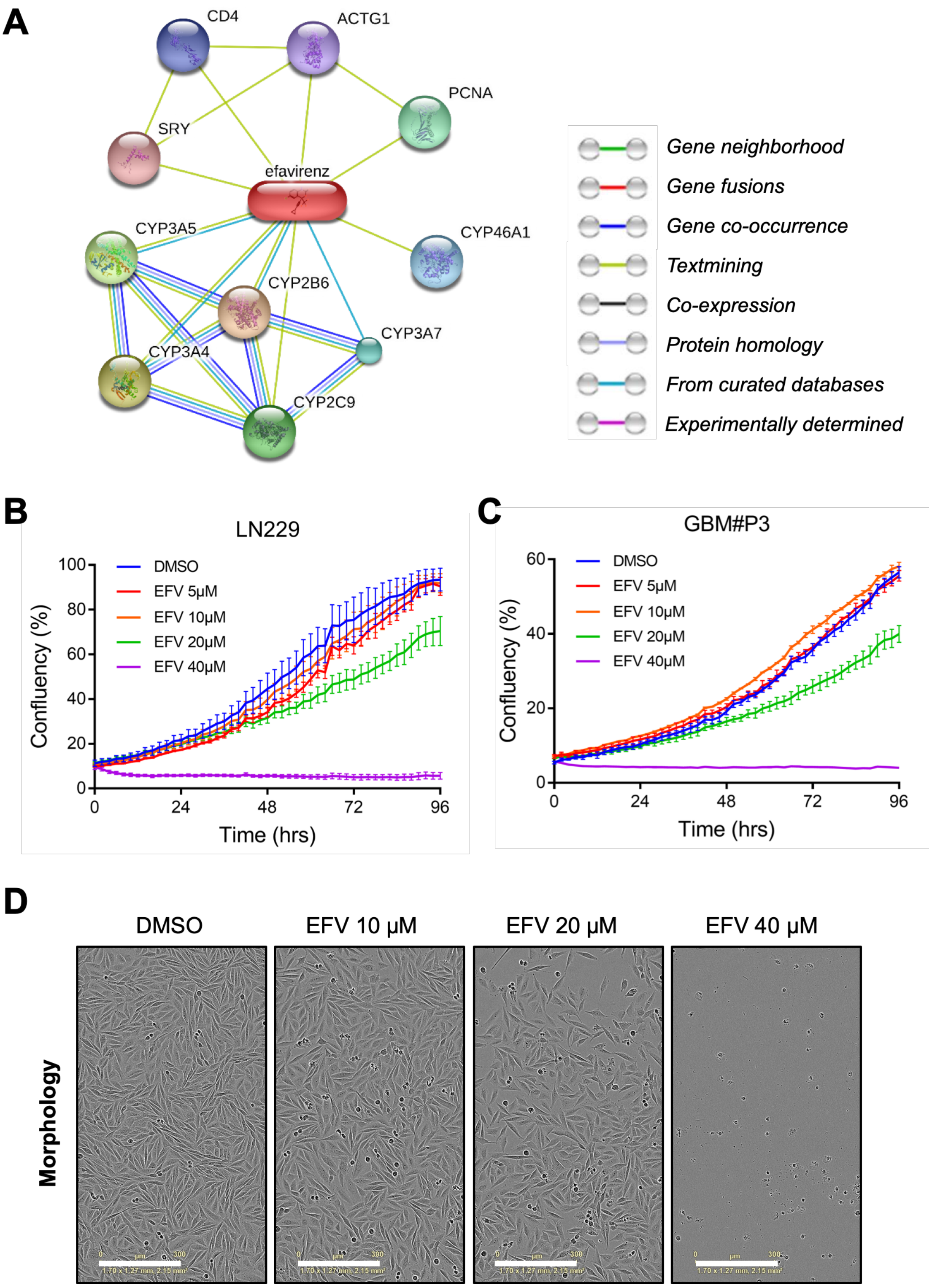

Figure S8 (A) Bioinformatic chemical-protein interaction network analysis of EFV obtained from STITCH. (B-C) Growth curves for proliferation of LN229 and GBM#P3 cells treated with EFV (0 - 40 μM). Cell proliferation was measured using the IncuCyte Proliferation Assay. Data are shown as the mean ± SEM. (D) Representative images of morphological differences in LN229 cells treated with EFV (0 – 40 μM) after 96 h relative to vehicle control. Scale bar = 300 μm

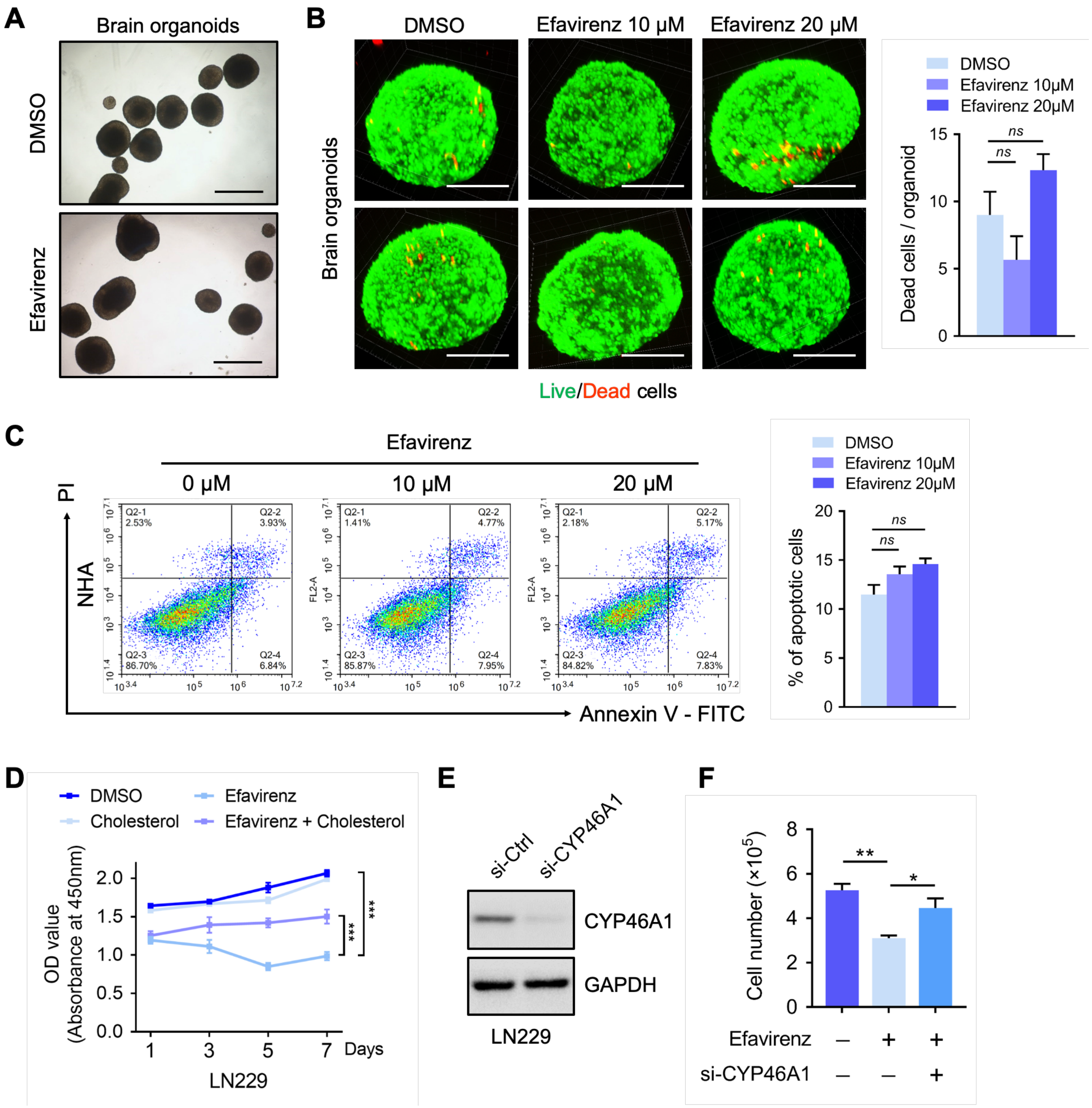

Figure S9 (A) Representative images of the morphology of brain organoids treated with DMSO or EFV (20 μM) for 72 h. Scale bar = 5 mm. (B) Representative images and quantification of immunofluorescence staining for live (green) and dead cells (red) in brain organoids after treatment with EFV (0 - 20 μM) for 72 h. Scale bar = 2 mm. Data are shown as the mean ± SEM (n = 3; one-way ANOVA). (C) Flow cytometry to detect annexin V-FITC and PI staining to assess apoptosis in NHA after treatment with EFV (0 - 20 μM) for 72 h. Data are shown as the mean ± SEM (n = 3; one-way ANOVA). (D) OD values from the CCK-8 assay plotted against time in days to generate cell growth curves for LN229 cells treated with DMSO or 20 μM EFV in the presence or absence of 0.5

μg/mL cholesterol. Data are shown as the mean ± SEM (*n* = 3). ****P* < 0.0001, ****P* = 0.0002 (one-way ANOVA). (E) Western blot analysis to confirm CYP46A1 knockdown in LN229 cells. (F) Cell viability of si-Ctrl or si-CYP46A1 LN229 cells after 3 days in the presence or absence of 20 μM EFV. Data are shown as the mean ± SEM (*n* = 3). ***P* = 0.006, **P* = 0.0448 (one-way ANOVA).

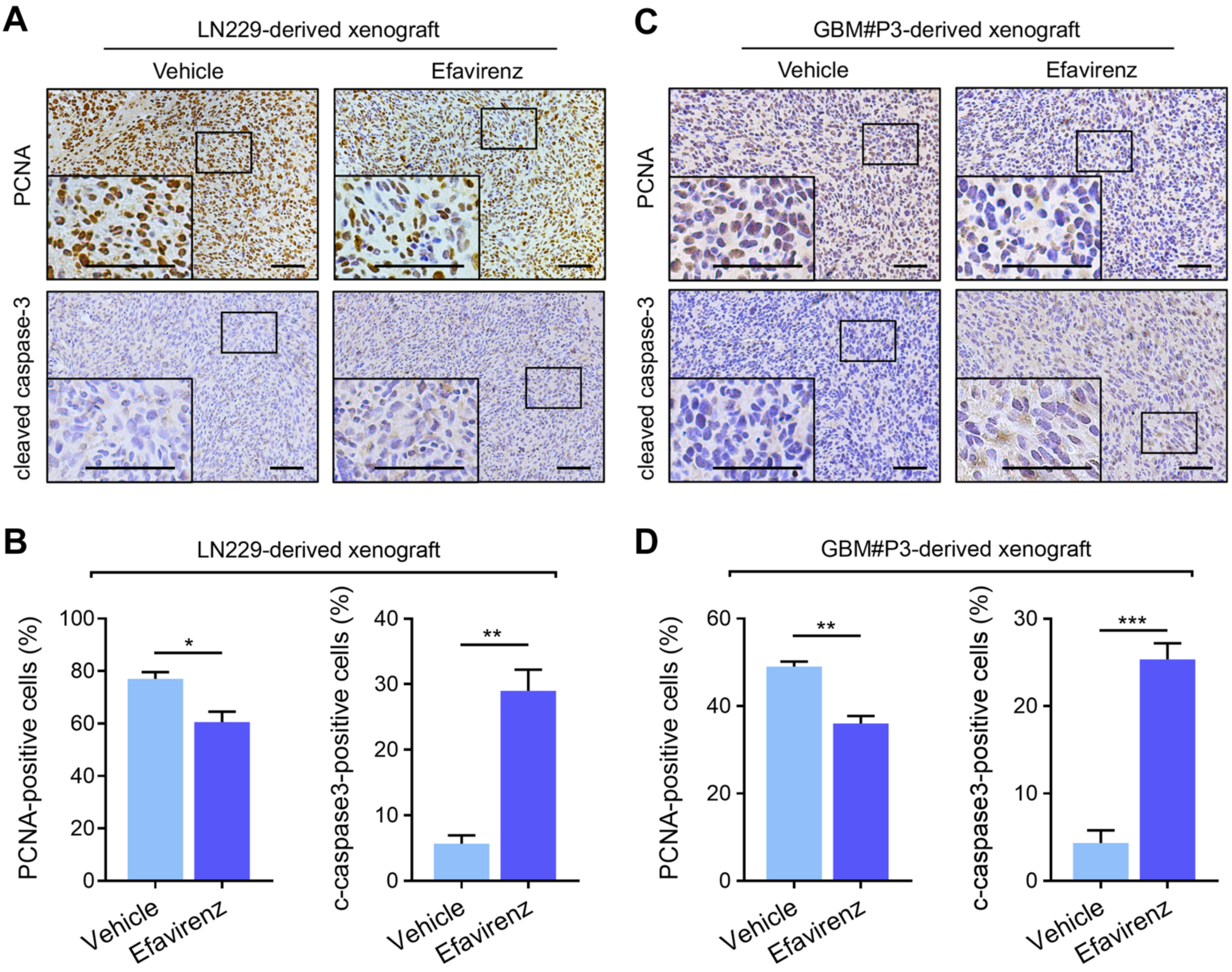

Figure S10 (A) IHC staining for PCNA and cleaved caspase-3 in sections from LN229 xenografts. Scale bar = 100 μm. (B) Graphic representation of the quantification of IHC staining in (A). (C) IHC staining for PCNA and cleaved caspase-3 in sections from GBM#P3 xenografts. Scale bar = 100 μm. (D) Graphic representation of the quantification of IHC staining in (C). Data are shown as the mean ± SEM. LN229: **P* = 0.0273, ***P* = 0.0026; GBM#P3: ***P* = 0.0034, ****P* = 0.0009. (two-sided Student's t-test).

Table S1.

TCGA

Variable	Univariate Cox Regression		Multivariate Cox Regression	
	HR (95 % CI)	*P*	HR (95 % CI)	*P*
Age<br>Increasing years	1.069 (1.058-1.080)	<0.001	1.035 (1.022-1.047)	<0.001
Gender<br>Female *vs* male	1.119 (0.680-1.842)	0.657		
WHO grade<br>High- *vs* low-	4.723 (3.821-5.837)	<0.001	2.012 (1.528-2.650)	<0.001
CYP46A1 expression<br>High *vs* low	**0.462 (0.603-2.541)**	**<0.001**	1.110 (0.812-1.517)	0.514
IDH status<br>Mutation *vs* wild-type	0.101 (0.076-0.135)	<0.001	0.337 (0.215-0.529)	<0.001
1p/19q status<br>Codel *vs* Non-codel	0.217 (0.137-0.344)	<0.001	0.543 (0.313-0.941)	0.029

CGGA

Variable	Univariate Cox Regression		Multivariate Cox Regression	
	HR (95 % CI)	*P*	HR (95 % CI)	*P*
Age<br>Increasing years	1.036 (1.022-1.051)	< 0.001	0.997 (0.980-1.015)	0.734
Gender<br>Female *vs* male	0.812 (0.579-1.137)	0.225		
WHO grade<br>High- *vs* low-	5.432 (3.793-7.781)	< 0.001	2.747 (1.763-4.280)	< 0.001
CYP46A1 expression<br>High *vs* low	**0.289 (0.206-0.409)**	**< 0.001**	**0.390 (0.262-0.581)**	**< 0.001**
IDH status<br>Mutation *vs* wild-type	0.257 (0.181-0.364)	< 0.001	0.374 (0.237-0.587)	< 0.001
Radiotherapy<br>Yes *vs* no	0.450 (0.312-0.649)	< 0.001	0.492 (0.340-0.713)	< 0.001
Chemotherapy<br>Yes *vs* no	0.719 (0.505-1.025)	0.068		

HR, hazards ratio; CI, confidence interval.

Table S2.

Table 1. STR profiles of LN229 cell line

	Allele1	Allele2
D3S1358	16	17
TH01	9.3	
D21S11	29	30
D18S51	13	15
Penta_E	7	16
D5S818	11	12
D13S317	10	11
D7S820	8	11
D16S539	12	
CSF1PO	12	
Penta_D	10	11
AMEL	x	
vWA	16	19
D8S1179	13	
TPOX	8	
FGA	23	

Figure 1. STR profiles of LN229 cell line

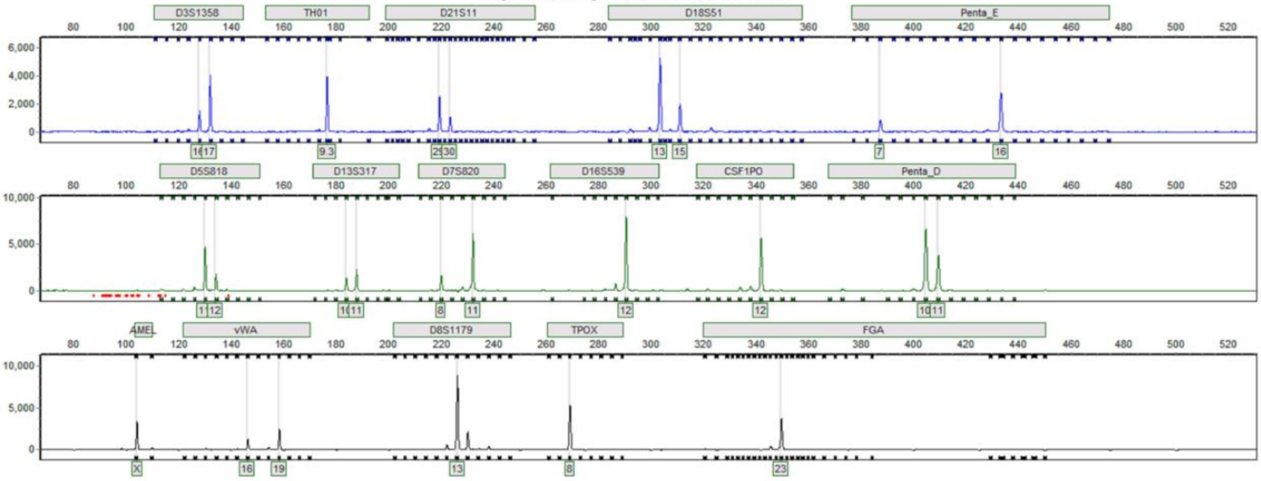

Table 1: STR profiles of GBM#P3

Sample No. GBM#P3	
Marker	Allele
D19S433	16.2
D5S818	10,11
D21S11	31.2,33.2
D18S51	12,15
D6S1043	12,18
AMEL	X,Y
D3S1358	14,16
D13S317	11,12
D7S820	14
D16S539	11,12
CSF1PO	10,12
Penta D	10,11
D2S441	11
vWA	16,17
D8S1179	14,15
TPOX	8
Penta E	5,15
TH01	9
D12S391	19,26
D2S1338	17,23
FGA	23,25

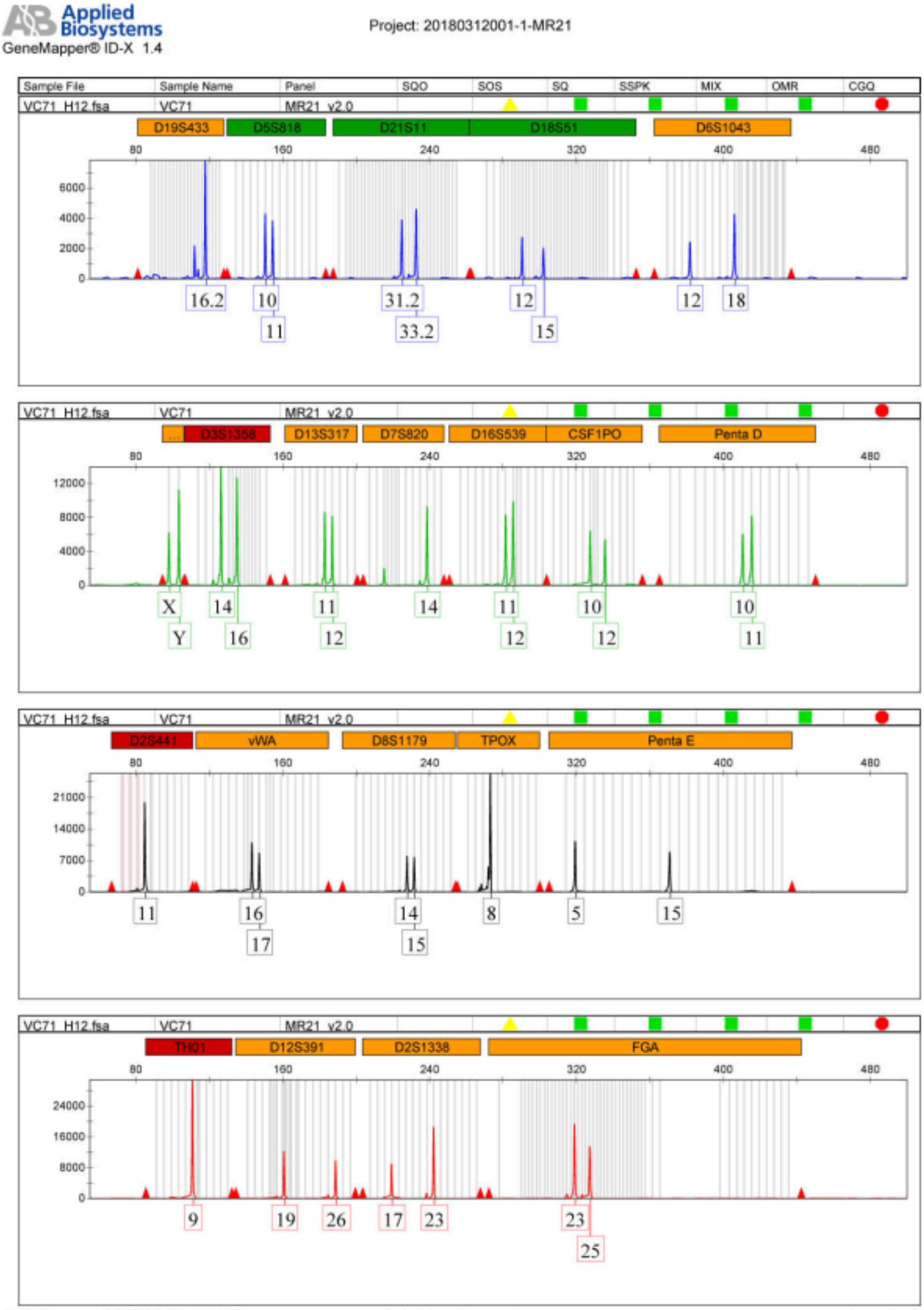

Figure 1. STR profiles of GBM#P3

Sample No：GBM#BG7	
Marker	Allele
D3S1358	15,17
TH01	9,9.3
D21S11	31.2,32.2
D18S51	12
Penta E	16,17
D5S818	12
D13S317	8
D7S820	10
D16S539	12,13
CSF1PO	11,13
Penta D	10,13
AMEL	X
vWA	14,19
D8S1179	14
TPOX	8
FGA	21,22
D19S433	12,13
D12S391	20,21
D6S1043	11,20
D2S1338	17,20
D1S1656	17.3

GeneMapper® ID-X 1.4

Project: 20181025001-HE21

Sample File	Sample Name	Panel	SQO	SOS	SQ	SSPK	MIX	OMR	CGQ
2018-CELL-281_B03.fsa	GBM#BG7	HGT_21G-v2.0							

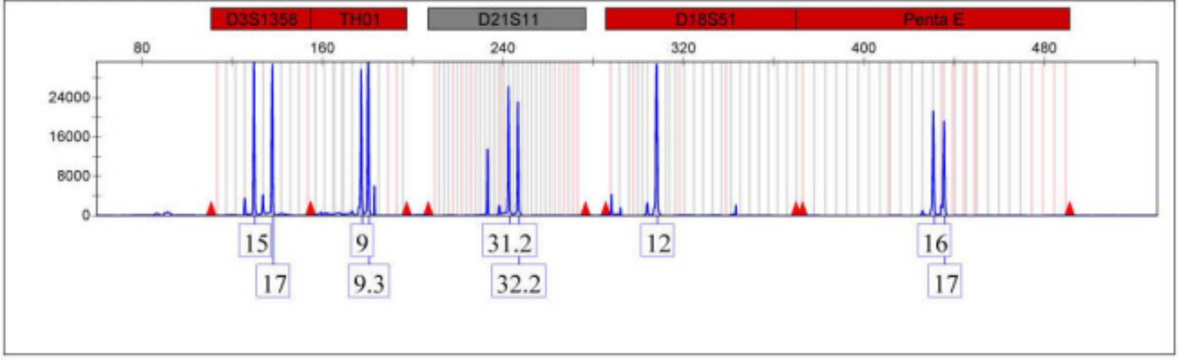

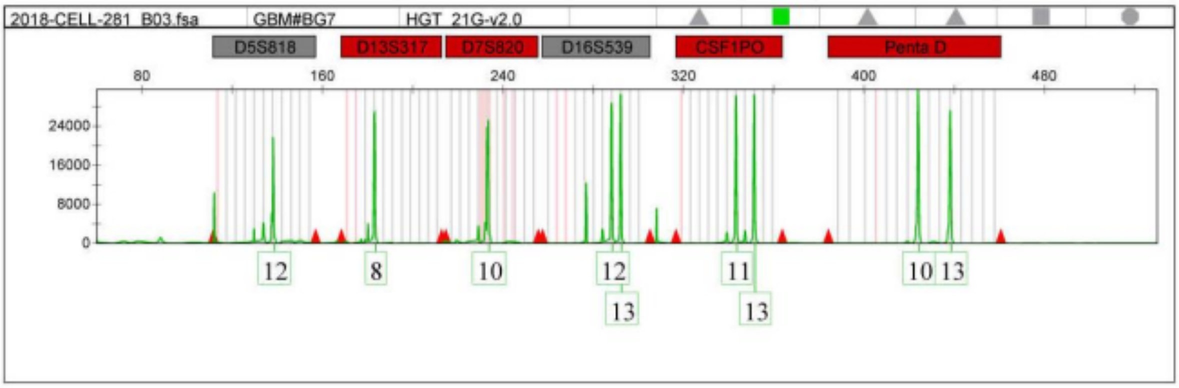

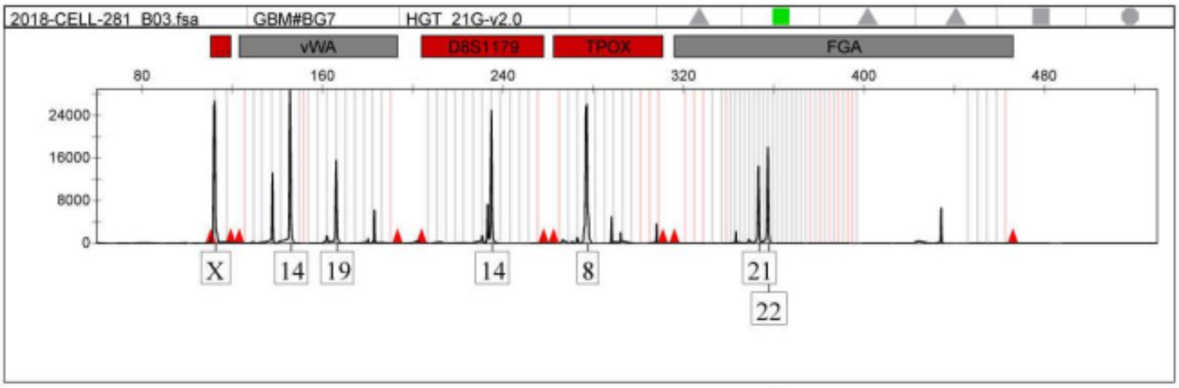

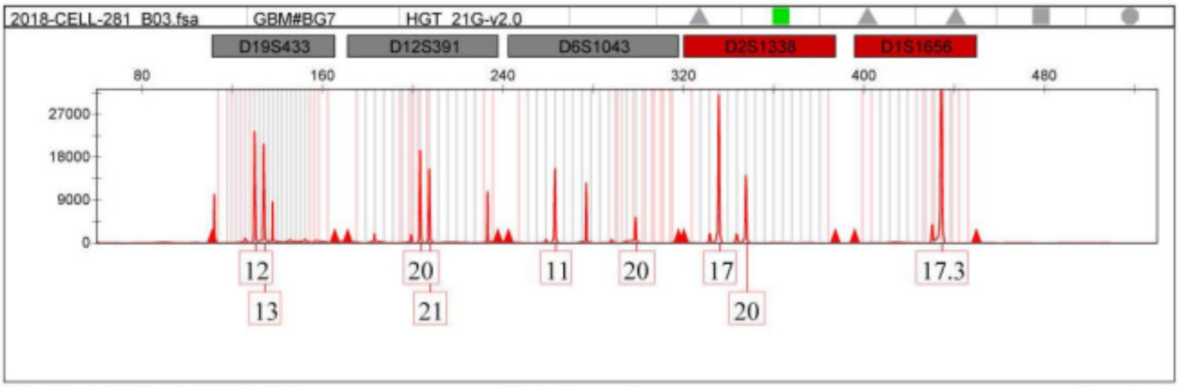

Sample No：GBM#BG5	
Marker	Allele
D3S1358	18,19
TH01	9
D21S11	29,32.2
D18S51	13,18
Penta E	7,11
D5S818	11,13
D13S317	11,13
D7S820	10
D16S539	10,11
CSF1PO	10,11
Penta D	9,13
AMEL	X
vWA	15,16
D8S1179	8,10
TPOX	8
FGA	19,21
D19S433	14,15
D12S391	23
D6S1043	11,14
D2S1338	17,21
D1S1656	12

Applied Biosystems
GeneMapper® ID-X 1.4

Project: 20181025001-HE21

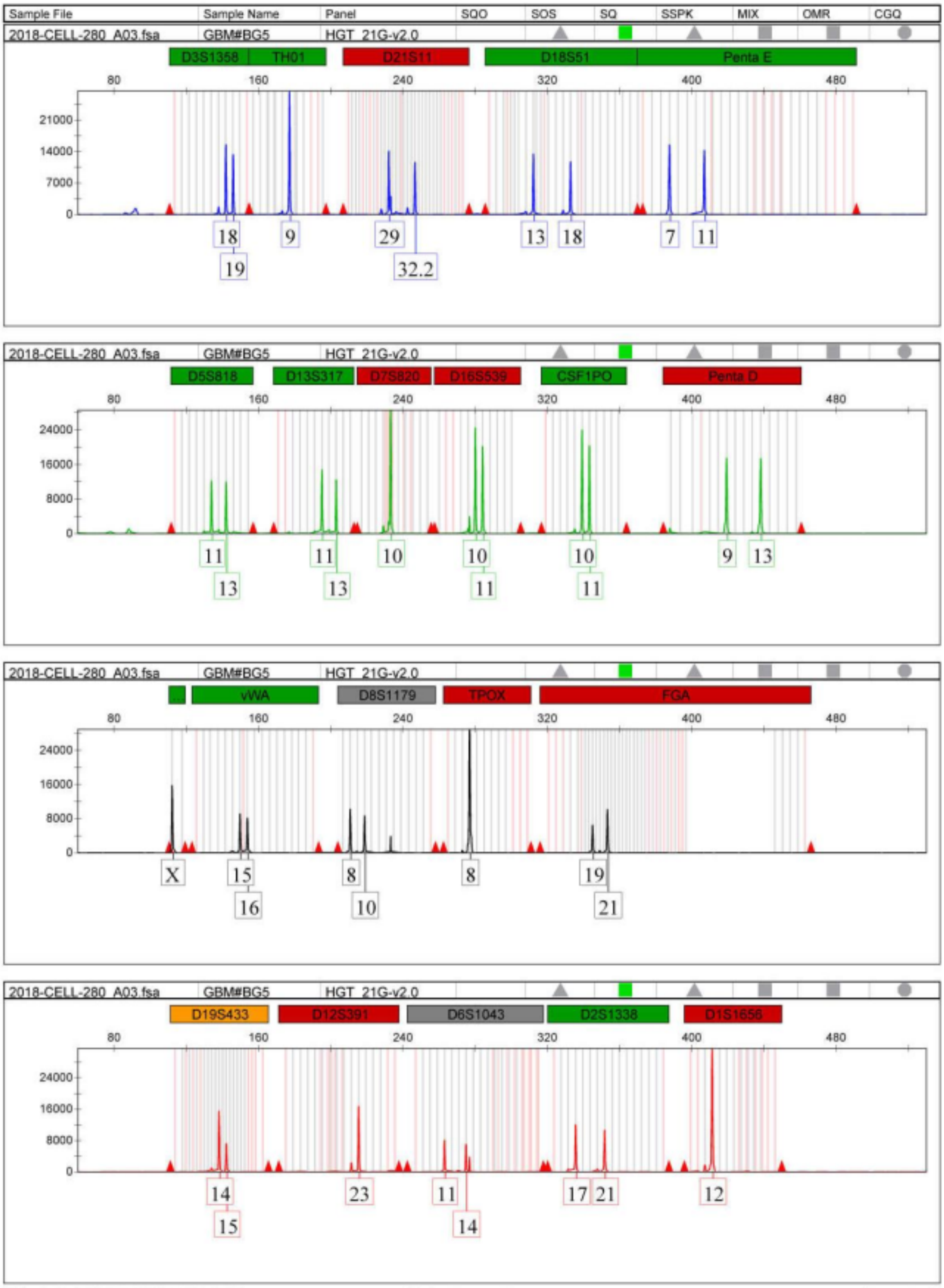

Table S3.

Gene	Forward primer	Reverse primer
CYP46A1	TAGGACACCTCCCCTGCTTT	AGGAACTTCTTAACCGACTCAGG
GAPDH	GGAGCGAGATCCCTCCAAAAT	GGCTGTTGTCATACTTCTCATGG
ABCA1	AGCCACCCTGGTTCCAA	CCACCTTCATCCCATCTCGG
ABCG1	ACAAAATCCGGGCAGAGAGG	AGACACCCACAAACCCAACG
IDOL	GCAGGCGACTGGGAATCATAG	CGGTTTCTCAGGTTTAGCCAT
APOE	GGACAGGGGGAGCCCTATAA	GTGATTGGCCTGTCGGCTC
HMGCR	CTGGGGAATTGTCACTTATGG	GAACTGTCGGGCTATTCAGG
FASN	AGTACACACCCAAGGCCAAG	GTGGATGATGCTGATGATGG
ACC1	TGACAGAGGAGGATGGTGTTC	GCTGAGTGGGTGATATGTGCT
SOX2	GCTGGGCGCCGAGTGGA	GGCGAGCGTTCATGTAGGTCTG
