# Supplementary material for: Therapeutic implications of altered cholesterol homeostasis mediated by loss of CYP46A1 in human glioblastoma
Source: EMBO Mol Med. 2019 Nov 28;12(1):e10924. doi: 10.15252/emmm.201910924 (PMC6949512; doi:10.15252/emmm.201910924)
Supplement: Supplementary file 2 — Expanded View Figures PDF [file EMMM-12-e10924-s002.pdf]

Expanded View Figures

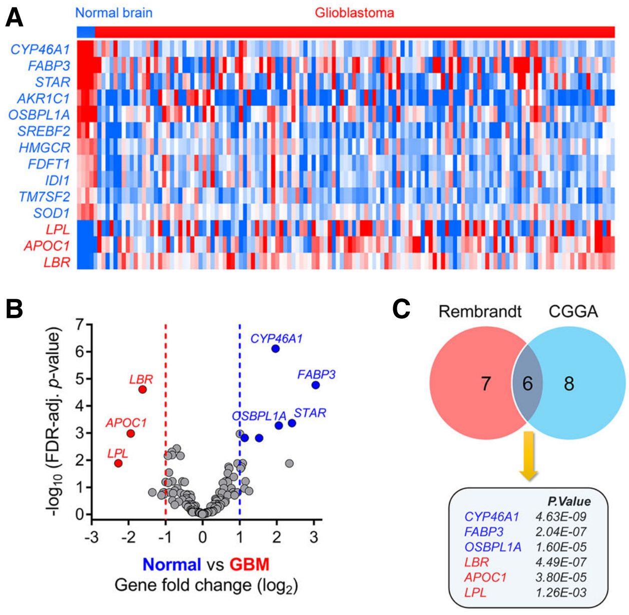

D

GENE	Regression Coefficient Value	Hazard Ratio	*P* value
APOBR	0.57	1.768	0.000042
CELA3A	1.893	6.64	0.000087
CYP46A1	-0.477	0.621	0.00013
MALL	0.431	1.539	0.00031
SREBF2	-0.755	0.47	0.0017
SORL1	-0.452	0.636	0.0019
NUS1	0.683	1.98	0.0049
NPC2	0.267	1.306	0.0073
CLN6	0.563	1.756	0.0098
CEL	0.797	2.219	0.01
TNFSF4	0.446	1.563	0.013
FDFT1	-0.388	0.678	0.016
APOC1	0.184	1.202	0.018
ABCA1	0.27	1.31	0.023
SOAT1	1.574	4.827	0.026
G6PC	-95	5.58E-42	0.028
LMF1	-0.1292	0.8788	0.035
LSS	1.594	4.924	0.039
FDX1	0.492	1.636	0.043
CYP51A1	-0.428	0.652	0.046

Figure EV1. Expression of *CYP46A1* correlates strongly with malignant features in GBM.

A Heatmap of the differentially expressed cholesterol-related genes between normal brain tissues ($n = 5$) and glioblastomas ($n = 128$) from the CGGA dataset. Gene expression values are z-transformed and coloured red for high expression and blue for low expression, as indicated in the scale bar.

B Volcano plot showing the fold change (log2) in cholesterol-related gene levels based on GBM versus normal brain tissue samples.

C Venn plot showing the significantly dysregulated genes both in Rembrandt and in CGGA datasets.

D The top 20 cholesterol-related genes significantly associated with overall survival in GBM patients from the CGGA dataset.

© 2019 The Authors

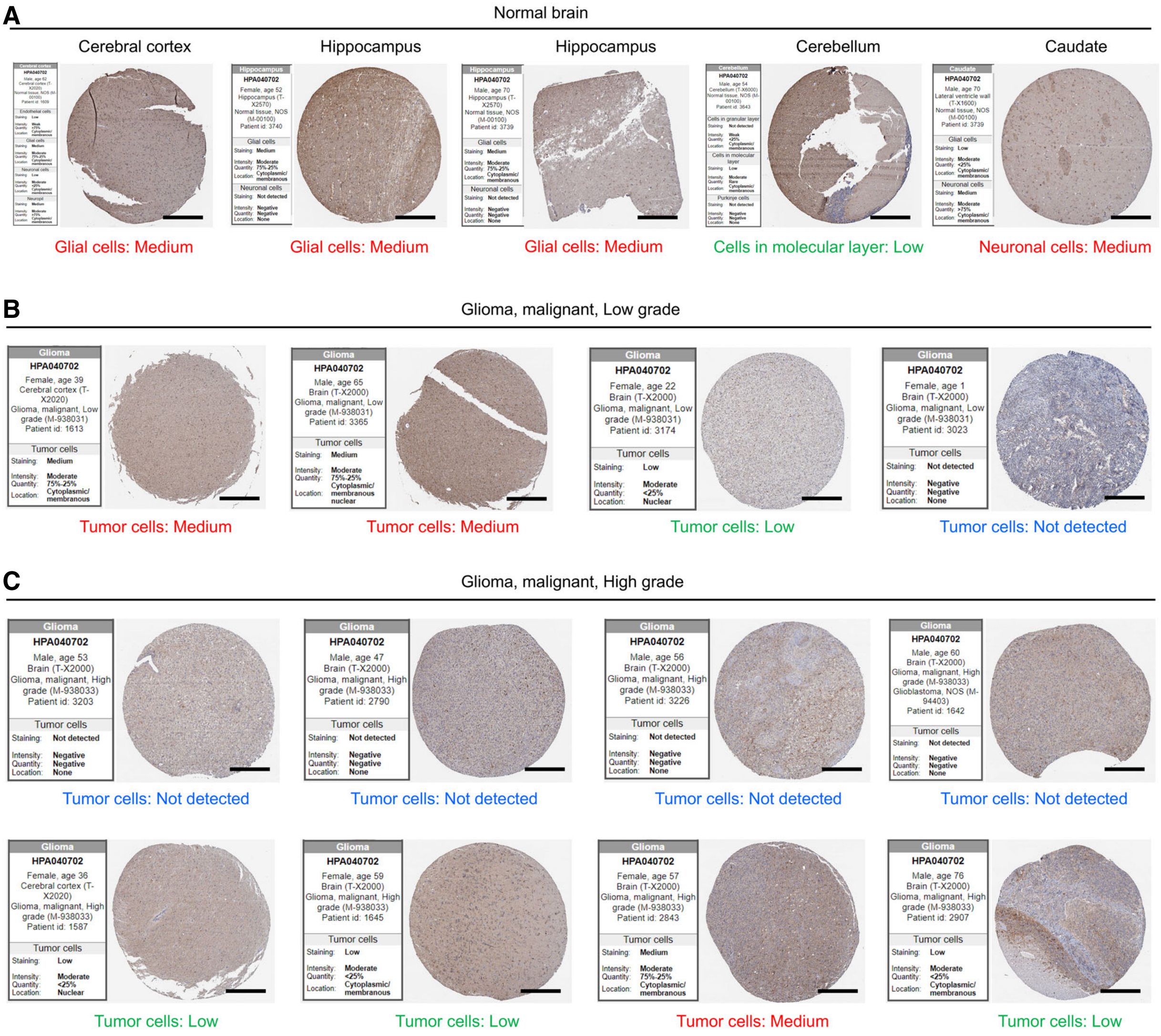

Figure EV2. Decreased expression of CYP46A1 in high-grade gliomas in comparison with normal brain.

A–C Representative images of IHC staining of CYP46A1 in normal brain and glioma tissues with quantification provided by the Human Protein Atlas (n = 5 for cerebellum, cerebral cortex and hippocampus, n = 4 for low-grade glioma and n = 8 for high-grade glioma). Scale bar = 200 μm. The scores of stained tissues were estimated for each cell type, including total staining (not detected to high), intensity (negative to strong), quantity (negative to > 75%) and sub-cellular locations of positive staining.

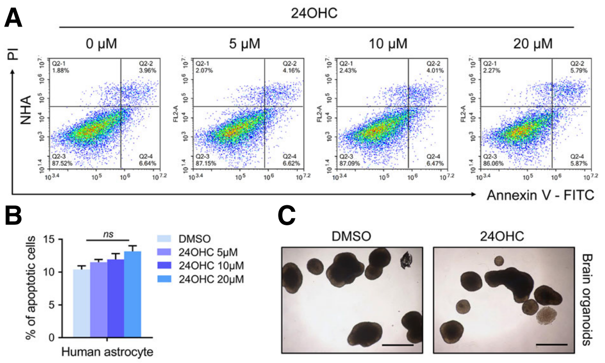

Figure EV3. Normal human astrocytes (NHAs) and brain organoids are resistant to exogenous 24OHC treatment.

A, B Flow cytometry to detect Annexin V-FITC and PI staining to assess apoptosis in NHA after treatment with 24OHC (0–20 μM) for 72 h. Data are shown as the mean ± SEM (n = 3). NS: not significant. Statistical significance was determined by one-way ANOVA.

C Representative images of the morphology of rat brain organoids treated with DMSO or 20 μM 24OHC for 72 h. Scale bar = 5 mm.

© 2019 The Authors
